# Universal Healthcare in the United States of America: A Healthy Debate

**DOI:** 10.3390/medicina56110580

**Published:** 2020-10-30

**Authors:** Gabriel Zieff, Zachary Y. Kerr, Justin B. Moore, Lee Stoner

**Affiliations:** 1 Department of Exercise and Sport Science, University of North Carolina at Chapel Hill, Chapel Hill, NC 27599, USA; zkerr@email.unc.edu (Z.Y.K.); dr.l.stoner@gmail.com (L.S.); 2 Department of Implementation Science, Wake Forest School of Medicine, Winston-Salem, NC 27157, USA; jusmoore@wakehealth.edu

**Keywords:** chronic disease, health insurance, socio–economic status, obesity, diabetes, hypertension, health promotion, universal healthcare

## Abstract

This commentary offers discussion on the pros and cons of universal healthcare in the United States. Disadvantages of universal healthcare include significant upfront costs and logistical challenges. On the other hand, universal healthcare may lead to a healthier populace, and thus, in the long-term, help to mitigate the economic costs of an unhealthy nation. In particular, substantial health disparities exist in the United States, with low socio–economic status segments of the population subject to decreased access to quality healthcare and increased risk of non-communicable chronic conditions such as obesity and type II diabetes, among other determinants of poor health. While the implementation of universal healthcare would be complicated and challenging, we argue that shifting from a market-based system to a universal healthcare system is necessary. Universal healthcare will better facilitate and encourage sustainable, preventive health practices and be more advantageous for the long-term public health and economy of the United States.

## 1. Introduction

Healthcare is one of the most significant socio–political topics in the United States (U.S.), and citizens currently rank “healthcare” as the most important issue when it comes to voting [1]. The U.S. has historically utilized a mixed public/private approach to healthcare. In this approach, citizens or businesses can obtain health insurance from private (e.g., Blue Cross Blue Shield, Kaiser Permanente) insurance companies, while individuals may also qualify for public (e.g., Medicaid, Medicare, Veteran’s Affairs), government-subsidized health insurance. In contrast, the vast majority of post-industrial, Westernized nations have used various approaches to provide entirely or largely governmentally subsidized, universal healthcare to all citizens regardless of socio–economic status (SES), employment status, or ability to pay. The World Health Organization defines universal healthcare as “ensuring that all people have access to needed health services (including prevention, promotion, treatment, rehabilitation and palliation) of sufficient quality to be effective while also ensuring that the use of these services does not expose the user the financial hardship” [2]. Importantly, the Obama-era passage of the Affordable Care Act (ACA) sought to move the U.S. closer to universal healthcare by expanding health coverage for millions of Americans (e.g., via Medicaid expansion, launch of health insurance marketplaces for private coverage) including for citizens across income levels, age, race, and ethnicity.

Differing versions of universal healthcare are possible. The United Kingdom’s National Health Services can be considered a fairly traditional version of universal healthcare with few options for, and minimal use of, privatized care [3]. On the other hand, European countries like Switzerland, the Netherlands, and Germany have utilized a blended system with substantial government and market-based components [4,5]. For example, Germany uses a multi-payer healthcare system in which subsidized health care is widely available for low-income citizens, yet private options—which provide the same quality and level of care as the subsidized option—are also available to higher income individuals. Thus, universal healthcare does not necessarily preclude the role of private providers within the healthcare system, but rather ensures that equity and effectiveness of care at population and individual levels are a reference and expectation for the system as a whole. In line with this, versions of universal healthcare have been implemented by countries with diverse political backgrounds (e.g., not limited to traditionally “socialist/liberal” countries), including some with very high degrees of economic freedom [6,7].

Determining the degree to which a nation’s healthcare is “universal” is complex and is not a “black and white” issue. For example, government backing, public will, and basic financing structure, among many other factors must be extensively considered. While an in-depth analysis of each of these factors is beyond the scope of this commentary, there are clear advantages and disadvantages to purely private, market-based, and governmental, universal approaches to healthcare, as well as for policies that lie somewhere in-between. This opinion piece will highlight arguments for and against universal healthcare in the U.S., followed by the authors’ stance on this issue and concluding remarks.

## 2. Argument against Universal Healthcare

Though the majority of post-industrial Westernized nations employ a universal healthcare model, few—if any—of these nations are as geographically large, populous, or ethnically/racially diverse as the U.S. Different regions in the U.S. are defined by distinct cultural identities, citizens have unique religious and political values, and the populace spans the socio–economic spectrum. Moreover, heterogenous climates and population densities confer different health needs and challenges across the U.S. [8]. Thus, critics of universal healthcare in the U.S. argue that implementation would not be as feasible—organizationally or financially—as other developed nations [9]. There is indeed agreement that realization of universal healthcare in the U.S. would necessitate significant upfront costs [10]. These costs would include those related to: (i) physical and technological infrastructural changes to the healthcare system, including at the government level (i.e., federal, state, local) as well as the level of the provider (e.g., hospital, out-patient clinic, pharmacy, etc.); (ii) insuring/treating a significant, previously uninsured, and largely unhealthy segment of the population; and (iii) expansion of the range of services provided (e.g., dental, vision, hearing) [10].

The cost of a universal healthcare system would depend on its structure, benefit levels, and extent of coverage. However, most proposals would entail increased federal taxes, at least for higher earners [4,11,12]. One proposal for universal healthcare recently pushed included options such as a 7.5% payroll tax plus a 4% income tax on all Americans, with higher-income citizens subjected to higher taxes [13]. However, outside projections suggest that these tax proposals would not be sufficient to fund this plan. In terms of the national economic toll, cost estimations of this proposal range from USD 32 to 44 trillion across 10 years, while deficit estimations range from USD 1.1 to 2.1 trillion per year [14].

Beyond individual and federal costs, other common arguments against universal healthcare include the potential for general system inefficiency, including lengthy wait-times for patients and a hampering of medical entrepreneurship and innovation [3,12,15,16]. Such critiques are not new, as exemplified by rhetoric surrounding the Clinton Administration’s Health Security Act which was labeled as “government meddling” in medical care that would result in “big government inefficiency” [12,15]. The ACA has been met with similar resistance and bombast (e.g., the “repeal and replace” right-leaning rallying cry) as a result of perceived inefficiency and unwanted government involvement. As an example of lengthy wait times associated with universal coverage, in 2017 Canadians were on waiting lists for an estimated 1,040,791 procedures, and the median wait time for arthroplastic surgery was 20–52 weeks [17]. Similarly, average waiting time for elective hospital-based care in the United Kingdom is 46 days, while some patients wait over a year (3). Increased wait times in the U.S. would likely occur—at least in the short term—as a result of a steep rise in the number of primary and emergency care visits (due to eliminating the financial barrier to seek care), as well as general wastefulness, inefficiency, and disorganization that is often associated with bureaucratic, government-run agencies.

## 3. Argument for Universal Healthcare

Universal healthcare in the U.S., which may or may not include private market-based options, offer several noteworthy advantages compared to exclusive systems with inequitable access to quality care including: (i) addressing the growing chronic disease crisis; (ii) mitigating the economic costs associated with said crisis; (iii) reducing the vast health disparities that exist between differing SES segments of the population; and (iv) increasing opportunities for preventive health initiatives [18,19,20,21]. Perhaps the most striking advantage of a universal healthcare system in the U.S. is the potential to address the epidemic level of non-communicable chronic diseases such as cardiovascular diseases, type II diabetes, and obesity, all of which strain the national economy [22,23]. The economic strain associated with an unhealthy population is particularly evident among low SES individuals. Having a low SES is associated with many unfavorable health determinants, including decreased access to, and quality of health insurance which impact health outcomes and life expectancies [24]. Thus, the low SES segments of the population are in most need of accessible, quality health insurance, and economic strain results from an unhealthy and uninsured low SES [25,26]. For example, diabetics with low SES have a greater mortality risk than diabetics with higher SES, and the uninsured diabetic population is responsible for 55% more emergency room visits each year than their insured diabetic counterparts [27,28]. Like diabetes, hypertension—the leading risk factor for death worldwide [29], has a much higher prevalence among low SES populations [30]. It is estimated that individuals with uncontrolled hypertension have more than USD 2000 greater annual healthcare costs than their normotensive counterparts [31]. Lastly, the incidence of obesity is also much greater among low SES populations [32]. The costs of obesity in the U.S., when limited to lost productivity alone, have been projected to equate to USD 66 billion annually [33]. Accessible, affordable healthcare may enable earlier intervention to prevent—or limit risk associated with—non-communicable chronic diseases, improve the overall public health of the U.S., and decrease the economic strain associated with an unhealthy low-SES.

### Preventive Initiatives within A Universal Healthcare Model

Beyond providing insurance coverage for a substantial, uninsured, and largely unhealthy segment of society—and thereby reducing disparities and unequal access to care among all segments of the population—there is great potential for universal healthcare models to embrace value-based care [4,20,34]. Value-based care can be thought of as appropriate and affordable care (tackling wastes), and integration of services and systems of care (i.e., hospital, primary, public health), including preventive care that considers the long-term health and economy of a nation [34,35]. In line with this, the ACA has worked in parallel with population-level health programs such as the Healthy People Initiative by targeting modifiable determinants of health including physical activity, obesity, and environmental quality, among others [36]. Given that a universal healthcare plan would force the government to pay for costly care and treatments related to complications resulting from preventable, non-communicable chronic diseases, the government may be more incentivized to (i) offer primary prevention of chronic disease risk prior to the onset of irreversible complications, and (ii) promote wide-spread preventive efforts across multiple societal domains. It is also worth acknowledging here that the national public health response to the novel Coronavirus-19 virus is a salient and striking contemporary example of a situation in which there continues to be a need to expeditiously coordinate multiple levels of policy, care, and prevention.

Preventive measures lessen costs associated with an uninsured and/or unhealthy population [37]. For example, investing USD 10 per person annually in community-based programs aimed at combatting physical inactivity, poor nutrition, and smoking in the U.S. could save more than USD 16 billion annually within five years, equating to a return of USD 5.60 for every dollar spent [38]. Another recent analysis suggests that if 18% more U.S. elementary-school children participated in 25 min of physical activity three times per week, savings attributed to medical costs and productivity would amount to USD 21.9 billion over their lifetime [39]. Additionally, simple behavioral changes can have major clinical implications. For example, simply brisk walking for 30 min per day (≥15 MET-hours/week) has been associated with a 50% reduction in type II diabetes [40]. While universal healthcare does not necessarily mean that health policies supporting prevention will be enacted, it may be more likely to promote healthy (i) lifestyle behaviors (e.g., physical activity), (ii) environmental factors (e.g., safe, green spaces in low and middle-income communities), and (iii.) policies (e.g., banning sweetened beverages in public schools) compared to a non-inclusive system [34,35,36].

Nordic nations provide an example of inclusive healthcare coupled with multi-layered preventive efforts [41]. In this model, all citizens are given the same comprehensive healthcare while social determinants of health are targeted. This includes “mobilizing and coordinating a large number of players in society,” which encourages cooperation among “players” including municipal political bodies, voluntary organizations, and educational institutions [41]. Developmental and infrastructural contributions from multiple segments of society to a healthcare system may also better encourage government accountability compared to a system in which a select group of private insurers and citizens are the only “stakeholders.” Coordinated efforts on various non-insurance-related fronts have focused on obesity, mental health, and physical activity [41]. Such coordinated efforts within the Nordic model have translated to positive health outcomes. For example, the Healthcare Access and Quality (HAQ) Index provides an overall score of 0–100 (0 being the worst) for healthcare access and quality across 195 countries and reflects rates of 32 preventable causes of death. Nordic nations had an average HAQ score of 95.4, with four of the five nations achieving scores within the top 10 worldwide [42]. Though far more heterogenous compared to Nordic nations, (e.g., culturally, geographically, racially, etc.), the U.S. had a score of 89 (29th overall) [42]. To provide further context, other industrialized nations, which are more comparable to the U.S. than Nordic nations, also ranked higher than the U.S. including Germany (92, 19th overall), Canada (94, 14th overall), Switzerland (96, 7th overall), and the Netherlands (96, 3rd overall) [42].

## 4. Conclusions

Non-inclusive, inequitable systems limit quality healthcare access to those who can afford it or have employer-sponsored insurance. These policies exacerbate health disparities by failing to prioritize preventive measures at the environmental, policy, and individual level. Low SES segments of the population are particularly vulnerable within a healthcare system that does not prioritize affordable care for all or address important determinants of health. Failing to prioritize comprehensive, affordable health insurance for all members of society and straying further from prevention will harm the health and economy of the U.S. While there are undoubtedly great economic costs associated with universal healthcare in the U.S., we argue that in the long-run, these costs will be worthwhile, and will eventually be offset by a healthier populace whose health is less economically burdensome. Passing of the Obama-era ACA was a positive step forward as evident by the decline in uninsured U.S. citizens (estimated 7–16.4 million) and Medicare’s lower rate of spending following the legislation [43]. The U.S. must resist the current political efforts to dislodge the inclusive tenets of the Affordable Care Act. Again, this is not to suggest that universal healthcare will be a cure-all, as social determinants of health must also be addressed. However, addressing these determinants will take time and universal healthcare for all U.S. citizens is needed now. Only through universal and inclusive healthcare will we be able to pave an economically sustainable path towards true public health.
